# Celebrating 50 Years of Excellence: The Legacy of Cardiovascular Surgery in Brazil and the Role of BJCVS

**DOI:** 10.21470/1678-9741-2024-0992

**Published:** 2024-04-03

**Authors:** Andreia C. Feitosa do Carmo, Camila Sáfadi Alves Gonçalves, Paulo Roberto B. Evora

**Affiliations:** 1 Hospital São Paulo, Escola Paulista de Medicina da Universidade Federal de São Paulo, (EPM – UNIFESP), São Paulo, SP, Brazil; 2 Sociedade Brasileira de Cirurgia Cardiovascular, São Paulo, SP, Brazil; 3 Faculdade de Medicina de Ribeirão Preto da Universidade de São Paulo (FMRP-USP), Ribeirão Preto, SP, Brazil

The heart is not just the engine of life; it is the essence of our existence, with each beat promising a new moment. Beyond its primary function of pumping blood, the heart is the core of our health, propelling life by circulating oxygen and essential nutrients throughout the body. Caring for the heart is therefore vital to sustaining a long and fulfilling life—a task embraced with passion by modern medicine, which ceaselessly seeks to understand and cure heart diseases.

Over the past century, cardiology has evolved significantly. From William Harvey’s 17^th^-century descriptions of blood circulation to today’s advanced technologies such as sphygmomanometry, electrocardiography, and radiology, cardiology has established itself as an essential medical specialty^[1]^. In the early 20^th^ century, cardiac surgery faced major challenges, with few treatment options available for serious heart diseases. Modern heart surgery began to take shape with the development of cardiopulmonary bypass machines and innovative techniques. In the United States, pioneers like John Gibbon and Clarence Walton Lillehei led significant advances. Gibbon performed the first successful surgery using a heart-lung machine in 1953, marking a milestone in medical history^[2]^. These advances laid the foundation for what has become one of the most dynamic and innovative areas of medicine.

While cardiology was advancing rapidly in the United States and Europe, Brazil was not far behind. The creation of the Sociedade Brasileira de Cirurgia Cardiovascular (SBCCV) in 1958 was a crucial step in consolidating cardiac surgery in the country. Founded by visionaries such as Euryclides Zerbini and Adib Jatene, SBCCV has become a fundamental pillar for the development of cardiology in Brazil, integrating international scientific knowledge with rich local clinical experience. Since then, the society has been essential in training highly qualified specialists and promoting significant innovations in the area^[3]^.

In 1986, the creation of the Brazilian Journal of Cardiovascular Surgery (BJCVS) marked a new chapter in the scientific dissemination and continuing education of cardiovascular surgery specialists. BJCVS quickly established itself as an indispensable platform for sharing research and significant advances in the field, providing professionals with a valuable resource for constant updating on techniques, discoveries, and emerging trends in cardiovascular medicine^[3]^. This publication has played a crucial role in the continuing education of specialists, reflecting Brazil's dedication to excellence in cardiac surgery.

The history of Brazilian heart surgery is a saga of pioneering, hard work, and success. From the first procedures performed almost simultaneously in major international centers to the introduction of innovative techniques such as beating-heart coronary artery bypass grafting and the use of bilateral internal thoracic artery grafts, Brazilian surgeons have been global leaders in the area. These contributions have not only improved surgical outcomes in Brazil but also had a significant impact worldwide, demonstrating the ability of Brazilian professionals to overcome challenges and achieve extraordinary milestones^[2]^.

The evolution of cardiac surgery in Brazil is closely intertwined with the history of renowned institutions such as the Heart Institute (InCor), which performed over 71,000 procedures between 1984 and 2007. This commitment to innovation and excellence has established Brazil as a leader in cardiovascular medicine, with referral centers that attract patients from around the world. The hard work and dedication of pioneers like Hugo Felipozzi, who performed the first open-heart surgery in Brazil in 1955, and Euryclides Zerbini, who performed the first heart transplant in Latin America in 1968, are clear examples of this successful trajectory^[2,3]^. In recent years, Brazilian cardiac surgery has continued to evolve, incorporating new technologies and advanced techniques.

Notably, studies on methylene blue stand out in the pages of BJCVS, exploring its potential use as a therapeutic agent in cases of circulatory shock and septicemia, as well as its role in myocardial protection during cardiac surgery procedures^[4]^. Such research is essential for understanding molecular mechanisms and identifying more effective treatments^[5]^.

Genomics is another field prominently featured in BJCVS, reflecting the revolution in understanding and treating heart disease at the genetic level. The journal frequently publishes studies on how genetic variations influence responses to cardiac therapies and disease risk, driving personalized medicine. For example, investigations into the impact of genetic variants on the efficacy of anticoagulants or predisposition to cardiomyopathies are commonly explored, providing valuable insights for clinical practice.

The future of cardiac surgery in Brazil is promising, marked by technological advances, international collaboration, and the exceptional skills of our professionals. To achieve this bright future, continued investment in the training and ongoing professional development of cardiac surgeons is essential. Comprehensive and up-to-date medical residency programs, fellowships at international reference centers, and participation in cutting-edge conferences are key to keeping our professionals at the forefront of innovations in the field^[3]^.

Realistic simulation in laboratories and high-fidelity virtual environments has become an essential ally in training for new techniques and preparing for challenging situations before entering the operating room. These simulation environments allow surgeons to practice complex procedures in a controlled setting that faithfully mimics human anatomy and physiology. By using sophisticated mannequins and virtual reality technologies, doctors can experience a wide range of clinical situations, from routine surgeries to critical emergencies, without risking real patients. This innovative approach to training offers several advantages. First, it allows surgeons to refine their technical skills repeatedly, free from the pressures of the actual surgical environment. They can practice sutures, resections, and other delicate procedures, gaining confidence and competence before performing them on patients. In addition, simulated scenarios can be tailored to address the specific needs of each surgeon, targeting areas for improvement and strengthening existing skills.

Another significant advantage is the ability to train entire healthcare teams collaboratively. Team simulation promotes effective communication, coordination, and quick decision-making, essential for success in real surgeries. Team members, including surgeons, anesthesiologists, nurses, and perfusionists, can work together in a safe environment to solve complex problems and improve their interactions in high-pressure situations. Furthermore, realistic simulation aids in identifying and correcting errors before they occur in real situations, thereby enhancing patient safety. Through exposure to challenging scenarios and learning to manage unexpected complications, surgeons develop the resilience necessary to handle medical emergencies. Studies indicate that simulation training can substantially reduce the rate of medical errors and improve surgical outcomes. Therefore, investing in advanced simulation technologies is imperative for training the cardiac surgeons of the future. Simulation not only enhances technical skills but also strengthens professionals’ confidence, ensuring they are prepared to face the complex and dynamic challenges of operating rooms. With this rigorous preparation, surgeons are better equipped to provide high-quality care and improve patient outcomes^[1]^.

In addition to technical excellence, it is vital for Brazilian cardiac surgeons to cultivate ethics and professional responsibility. Empathy and compassion are key to providing humane and supportive care, allowing surgeons to connect with their patients, understand their concerns, and provide the best care possible. Collaborating effectively with other healthcare professionals is also crucial to ensuring comprehensive, high-quality care^[3]^.

Investing in continuing education and the development of technical and interpersonal skills is crucial for unlocking a promising future in Brazilian cardiac surgery. Through an unwavering commitment to excellence, multidisciplinary collaboration, adaptability to innovation, and the cultivation of ethics and compassion, we will build a legacy of quality of life and well-being for patients, elevating Brazilian heart surgery to a level of global excellence^[3]^.

The trajectory of cardiac surgery in Brazil is a testament to the human capacity to overcome challenges and achieve extraordinary accomplishments. From early pioneers to the latest technological advancements, every step of this journey marks a milestone in the relentless effort to save lives and improve patient well-being. Celebrating 50 years of excellence honors the legacy of those who came before us and inspires us to look to the future with hope and determination, confident that the pursuit of innovation and excellence will continue to guide our path^[2]^.
